# Editors’ Book Table

**Published:** 1872-10

**Authors:** 


### Editors' Book Table.

[Note.—All works reviewed in the columns of the Chicago Medical Journal may be found in the extensive stock of W. B. Keen, Cooke & Co., whose catalogue of Medical Books will be sent to any address upon request.]

BOOKS RECEIVED.

*The Physiology of Man. Vol. IV. Nervous System.* By Austin Flint, Jr., M.D.

The publication of this volume of the author's great work on human physiology, constitutes a new era for the American student, which will be hailed with gladness by every one desirous of substituting accurate scientific data as the bases of his practical deductions, for the vague, loose, and altogether too uncertain physiological knowledge which has hitherto constituted the foundations for practice of too many of our professional brethren.

Dr. Flint, from the beginning of his professional career, has been an earnest student and worker and an original thinker, and it is by such, and by such only, that physiology, especially,can be advanced. His previous contributions to physiological science have placed the medical profession under a deep and lasting obligation, and this, the last though by no means the least, only intensifies it.

Until within comparatively a few years the physiology of the nervous system has been a terra incognita to the whole profession, and even now the land is still not too well known to the majority, so that the author is quite right in considering this, his last, as the most important of all his labors.

We have already credited the author with originality as a thinker, he should also be recognized as an original reader. The bibliography of the nervous system has been most carefully studied in the original publications, from Sir Charles Bell's Monograph in 1811, which may be said to be the beginning of nervous physiology, down to Hammond, the author's accomplished coadjutor. With regard to the former our author implicates him in the guilt of plagiarism from Magendie. We had been under the impression that the controversy, concerning the claim of priority of discovery, between these, the fathers of English and French physiology respectively, had been hitherto considered a drawn battle. Our American authority decides in favor of the illustrious Frenchman. Did time permit, we should take much pleasure in extending this brief "notice" into an elaborate review of this valuable work, calling attention to each chapter, indeed to almost every successive page, replete with the evidences of patient study, of laborious investigation, and of accurate analysis; but we must unwillingly omit the grateful task and content ourselves with simply urging each one of our readers to "take a new departure in physiology" from a careful study of the book. H.

*The Principles and Practice of Surgery.* By FRANK HASTINGS HAMILTON, A.M., M.D., LL.D., Professor of the Practice of Surgery, etc., in Bellevue Hospital Medical College; Visiting Surgeon to Bellevue Hospital, etc., etc. Illustrated with 467 engravings on wood. New York: William Wood & Co., 27 Great Jones Street. 1872. Pp. 943.

We predict for this book a high place as a standard authority for both practitioners and students.

*On the Functional Diseases of the Renal, Urinary and Reproductive Organs, with a general review of Urinary Pathology.* By D. CAMPBELL, M.D., L.R.C.S., Edinburg, etc. Philadelphia: Lindsay & Blakiston. 1872. Pp. 300.

A lively, readable, entertaining and instructive *resume* of the subject.

*The Treatment of Syphilis with Subcutaneous Sublimate Injections.* By GEORGE LEWIN, Professor at the Fr. Wilh. University and Surgeon in Chief of the Syphilitic Wards and Skin Diseases of the Charite Hospital, Berlin. Translated by Carl Proegler, M.D., late Surgeon in the Prussian Service and in the U. S. Army, and E. H. Gale, M.D., late Surgeon U. S. Army. Philadelphia: Lindsay & Blakiston. 1872. Pp. 249.

PAMPHLETS.

*Medico-Legal Science.* By THAD. M. STEVENS, M.D., Indianapolis. Reprint from the Transactions of the Indiana State Medical Association. 1872.

*Transactions of the State Medical Society of Michigan for the year* 1872. Pp. 118.

